# Population structure and connectivity among coastal and freshwater Kelp Gull (*Larus dominicanus*) populations from Patagonia

**DOI:** 10.1371/journal.pone.0301004

**Published:** 2024-04-18

**Authors:** Tatiana Kasinsky, Natalia Rosciano, Juliana A. Vianna, Pablo Yorio, Leonardo Campagna

**Affiliations:** 1 Centro Para El Estudio de Sistemas Marinos, CONICET, Puerto Madryn, Chubut, Argentina; 2 Instituto de Investigaciones en Biodiversidad y Medio Ambiente, CONICET, Universidad Nacional del Comahue, San Carlos de Bariloche, Río Negro, Argentina; 3 Departamento de Ecología, Facultad de Ciencias Biológicas, Instituto para el Desarrollo Sustentable, Pontificia Universidad Católica de Chile, Santiago, Chile; 4 Millennium Institute Center for Genome Regulation (CRG), Millennium Institute of Biodiversity of Antarctic and Subantarctic Ecosystems (BASE), Millennium Nucleus of Patagonian Limit of Life (LiLi), Valdivia, Chile; 5 Wildlife Conservation Society Argentina, Ciudad Autónoma de Buenos Aires, Argentina; 6 Department of Ecology and Evolutionary Biology, Cornell University, Ithaca, NY, United States of America; 7 Fuller Evolutionary Biology Program, Cornell Lab of Ornithology, Ithaca, NY, United States of America; MARE – Marine and Environmental Sciences Centre, PORTUGAL

## Abstract

The genetic identification of evolutionary significant units and information on their connectivity can be used to design effective management and conservation plans for species of concern. Despite having high dispersal capacity, several seabird species show population structure due to both abiotic and biotic barriers to gene flow. The Kelp Gull is the most abundant species of gull in the southern hemisphere. In Argentina it reproduces in both marine and freshwater environments, with more than 100,000 breeding pairs following a metapopulation dynamic across 140 colonies in the Atlantic coast of Patagonia. However, little is known about the demography and connectivity of inland populations. We aim to provide information on the connectivity of the largest freshwater colonies (those from Nahuel Huapi Lake) with the closest Pacific and Atlantic populations to evaluate if these freshwater colonies are receiving immigrants from the larger coastal populations. We sampled three geographic regions (Nahuel Huapi Lake and the Atlantic and Pacific coasts) and employed a reduced-representation genomic approach to genotype individuals for single-nucleotide polymorphisms (SNPs). Using clustering and phylogenetic analyses we found three genetic groups, each corresponding to one of our sampled regions. Individuals from marine environments are more closely related to each other than to those from Nahuel Huapi Lake, indicating that the latter population constitutes the first freshwater Kelp Gull colony to be identified as an evolutionary significant unit in Patagonia.

## Introduction

Uncovering the mechanisms behind population genetic divergence is relevant for understanding both evolutionary processes and identifying evolutionary significant units for species in need of management and conservation strategies [1–4]. Moreover, the implementation of genomic techniques in the fields of ecology and conservation genetics has resulted in an increased ability to delimit populations, generate more complex models of demographic histories, and identify regions of the genome that confer important ecological functions and contribute to adaptation [4, 5].

Seabirds reproduce in discrete spatial populations (colonies); however, their ability to disperse over long distances [6] can lead to varying degrees of connectivity, resulting in metapopulation dynamics. However, several studies show some species have strong population structure, resulting from factors such as their non-breeding distribution, physical barriers to dispersal, the geographic distance between colonies, philopatry, and individual dispersal patterns [7].

The Kelp Gull (*Larus dominicanus*) is the most abundant and widely distributed gull species in the southern hemisphere, nesting in South America, Africa, New Zealand, Australia, sub-Antarctic islands, and the Antarctic Peninsula [8]. In Chile, they nest in at least 26 locations along the Pacific coast [9]. The estimated population in Chile comprises approximately ~8,200 breeding pairs, and colony sizes vary, ranging from just a few pairs to as many as 3,000, though due to the difficulty in surveying the geographically complex southern region, the actual numbers may potentially be higher [see 9]. Information regarding population trends in Chile is relatively scarce; however, preliminary data suggest an upward trend in the number of breeding pairs nesting on the rooftops of coastal cities [9]. On the other hand, in Argentina, the Kelp Gull breeds in both marine and freshwater environments [10], and its reproductive population in marine environments of Atlantic Patagonia was estimated at 106,200 breeding pairs distributed in 140 colonies [see 9]. Long-term monitoring of more than sixty colonies in northern Patagonia and the development of demographic models have shown high population connectivity and increases in numbers in recent decades, with demographic behaviors that vary depending on the breeding location [11, 12]. Reproductive Kelp Gull colonies in freshwater environments are limited to a few locations in Patagonia and Uruguay [9, 13, 14]. Little is known about demographic trends from freshwater populations in Argentinian Patagonia, however in the case of the colonies from Lake Nahuel Huapi, there has been an increase in the number of individuals breeding at one of the most important nesting sites, archipelago Islas de los Fósiles, within less than a decade (from 350 nests in 2012 to 575 nests in 2019).

**Fig 1:**
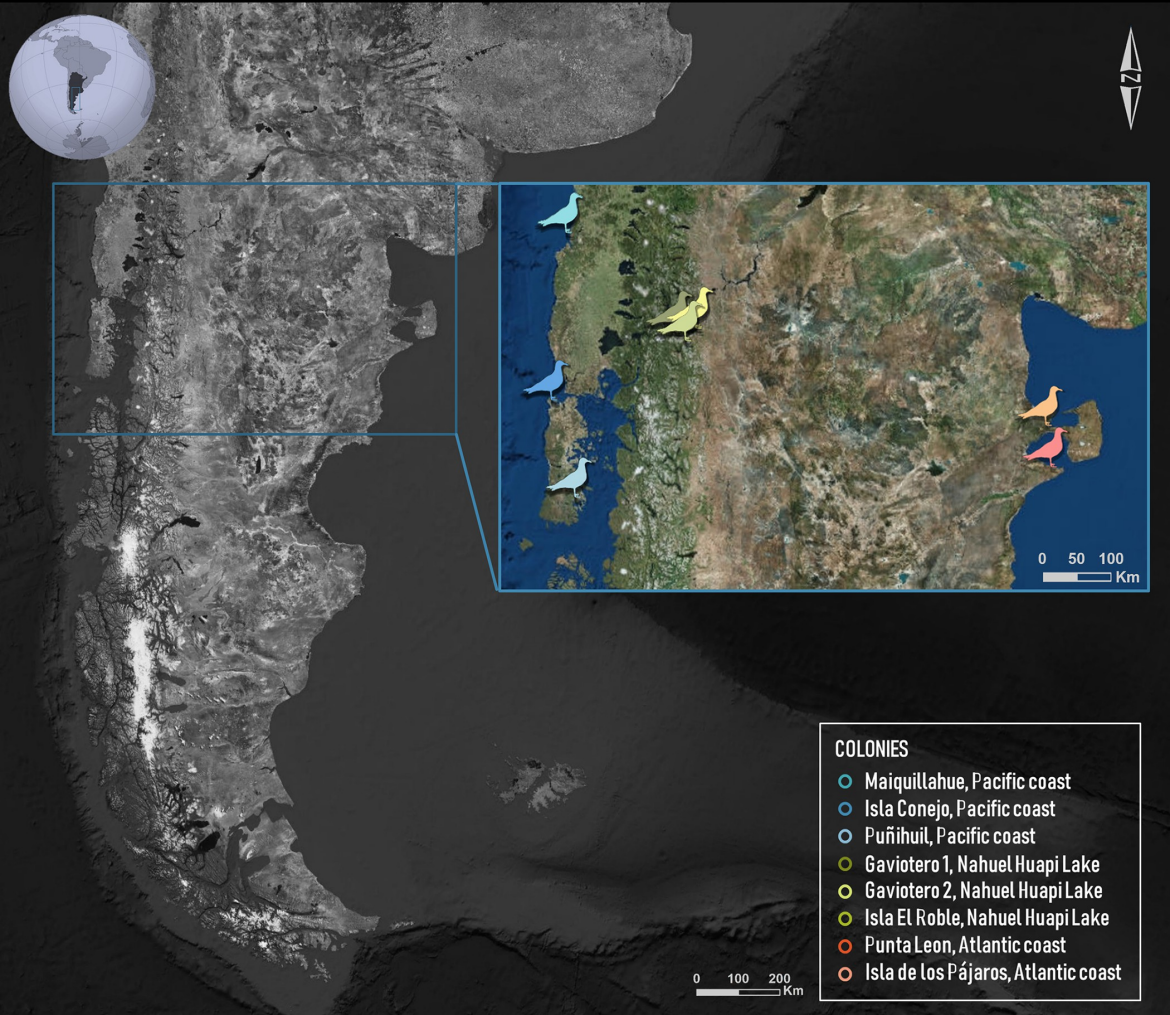
Map of the study area showing the Kelp Gull colonies from three regions. Pacific coast: Maiquillahue (n = 7), Isla Conejo (n = 3), Puñihuil (n = 4). Nahuel Huapi Lake: islets Gaviotero 1 (n = 36) and Gaviotero 2 (n = 32) (archipelago Islas de los Fósiles), Isla el Roble (n = 4). Atlantic coast: Isla de Los Pájaros (n = 15), Punta León (n = 16).

Studies conducted on gulls belonging to the genus *Larus* in various parts of the world have revealed genetic structure among populations, often following a pattern of isolation by distance [15–18]. Moreover, Herring Gull (*Larus argentatus*) populations from the Laurentian Great Lakes were found to be genetically distinct from their marine counterparts [19, 20], supporting the idea that reproductive Herring Gulls from the Great Lakes region constitute a closed and isolated system [21]. Genetic studies in the Kelp Gull show different degrees of population structure depending on their geographic scale, over a background of demographic expansion during the Holocene [16, 22–24]. However, the resolution provided by the genetic tools employed (mitochondrial DNA and microsatellite markers) and the scope of the sampling, particularly in the Patagonian region, may be underpowered to detect fine-scale population differences and adequately understand population connectivity. Studies carried out on other highly mobile seabirds have shown that the higher resolution of genomic data can reveal genetic structure that was previously undetected with mtDNA and microsatellite markers [25, 26]. In addition, Kelp Gull population studies in Argentina have focused on coastal regions, leaving an information gap regarding freshwater colonies. This study aims to provide information on the connectivity of the Lake Nahuel Huapi freshwater population with the closest coastal populations of the Pacific and Atlantic and to evaluate whether this freshwater population is an evolutionary significant unit. If the genetic structure in this species follows a pattern of isolation by distance, we would expect the Nahuel Huapi Lake individuals to show a stronger connection to those found along the Pacific coast, owing to the significantly shorter geographic distance than with respect to the Atlantic coast (see Fig 1). Despite the Andes Mountains potentially acting as a barrier between Nahuel Huapi Lake and the Pacific coast, it is conceivable that Kelp Gulls could be utilizing the interconnected network of lakes and rivers as natural conduits for genetic exchange between the two regions.

## Methods

### Fieldwork and sampling

We collected a total of 117 Kelp Gull samples (110 from blood and 7 from eggshells) across three different regions during the reproductive season (October to January of 2021 and 2022). The first region was located on the Pacific coast in Chile (PC, n = 14), where we sampled the colonies of Maiquillahue (39° 27’S, 73° 16’W), Puñihuil (41° 55’S, 74° 2’W), and Isla Conejo (42° 54’S, 73° 35’W). The second area was Nahuel Huapi Lake (NHL, n = 72), Argentina, where we collected samples from three colonies: the islets Gaviotero 1 and Gaviotero 2 (41°0’S, 71° 32’W) within the archipelago Islas de los Fósiles, and Isla el Roble (41°00’S, 71°27’W). Finally, the third region was the Atlantic coast in Argentina (AC, n = 31), where we sampled the colonies of Punta León (43°04’S, 64°29’W) and Isla de Los Pájaros (42°25’S, 64°31’W) (Fig 1). We captured the incubating adult from nests using a leg-noose trap or incubation traps [27] and subsequently collected a blood sample. In cases where it was impossible to capture adults, membranes were collected from newly hatched eggs, or blood (0.3–0.5 mL) was taken from non-related chicks (belonging to different nests and sectors of the colony). We conserved all samples in 96% ethanol until DNA extraction. We obtained the relevant permits for collecting and transporting samples from the corresponding national authorities in Argentina (Administración de Parques Nacionales DRPN N°1814, Dirección de Flora y Fauna Silvestre del Chubut Disp. N° 27/2022-DFyFS, Ministerio de Ambiente y Desarrollo Sostenible de la Argentina CE-2022-47128129-APN-DNBI#MAD, CE-2022-45430867-APN-DNBI#MAD) and Chile (Servicio Agrícola y Ganadero, La Corporación Nacional Forestal N° 5446/2022).

### Marker discovery and genotyping

We generated double digest restriction-site associated DNA markers (ddRAD tags) by first extracting genomic DNA using the DNeasy blood and tissue kit (Qiagen, Valencia, CA, USA). The DNA of each individual was digested with two restriction enzymes (*Sbf1* and *Msp1*; New England Biolabs; Ipswich, MA) and then ligated to specific adapters at the 5’ and 3’ ends for downstream bioinformatic processing. Adapter-ligated DNA from different individuals was combined into nine index groups and DNA fragments of between 400 and 700 base pairs were selected using the BluePippin size selection system (Sage Science, Beverly, MA, USA). The ddRAD protocol is described in detail in [28]. Index groups were combined into a single genomic library and sequencing took place on a lane of Illumina’s NextSeq500 platform at the Cornell Institute of Biotechnology (single-end x 150 bp mode), together with samples from another project.

We obtained an average of 453,047 ± 219,091 raw Illumina reads per individual. We evaluated read quality using FastQC version 0.11.6 (http://www.bioinformatics.babraham.ac.uk/projects/fastqc/). All sequences were trimmed to 112 bp using fastX trimmer [29] to remove lower-quality base pairs towards the 3’ end. Subsequently, reads that had any base calls with a quality score below Phred 10 (90% call accuracy) or with more than 20% of their base calls between 10 and 20 (90–99% call accuracy) were filtered using fastq_quality_filter (fastx-Toolkit). The reads were then demultiplexed and adapters were removed using the process_radtags module of the Stacks pipeline version 2.3e [30] to obtain files containing sequences specific to each individual. All sequences were trimmed to 105 bp after 5–7 bp inline barcodes were removed. Individual sequences were assembled using two methods implemented in the Stacks bioinformatics pipeline, a *de novo* pipeline, and a reference-based pipeline using the Herring Gull (*Larus smithsonianus*) reference genome obtained from www.ncbi.nlm.nih.gov (GCA_013400295.1_ASM1340029v1).

We aligned sequences to the Herring Gull reference genome using Bowtie 2 [31] with an average alignment rate of 89.17%. We subsequently assembled reads into markers using Stack’s gstacks module. This pipeline generated a catalog of 148,795 RAD loci with an effective per-sample coverage of 19.5 ± 9.0. After filtering out markers absent in more than 15% of individuals and with a minor allele count lower than 5 with the *populations* module, we retained and exported 6,569 SNPs. The *de novo* assembly was performed using ’ustacks/cstacks/sstacks’ as executed by the “denovo_map” pipeline and produced 2,735 SNPs after filtering. However, due to the similarity in the results from both assembly strategies, we decided to present those obtained from the reference-based assembly. We discarded five individuals with missing data higher than 25% (three from Gaviotero 1 and one from Gaviotero 2 in NHL, plus one from Isla de Los Pájaros in the Atlantic coast), retaining 112 individuals for all downstream analyses.

### Mitochondrial DNA amplification and sequencing

We obtained mitochondrial DNA (cytochrome c oxidase I, COI) from a subsample of five individuals from each population. COI amplification was conducted using polymerase chain reactions (PCRs) carried out as described in [32]. PCR products were cleaned by performing an Exo-SAP treatment (exonuclease -Exo- and shrimp alkaline phosphatase—SAP) before sequencing by incubating 10 ul of PCR product with 5 U of Exonuclease I and 0.5 U of SAP for 30 min at 37 C, followed by an inactivation step at 90 C for 10 min. Finally, the samples were Sanger sequenced bidirectionally at the Cornell Institute of Biotechnology with the same primers used for amplification (BirdF1 and COIBirdR2, [32]). Sequences were aligned using BioEdit v.7.0.5.3 [33], and alignments were checked manually. Relationships between haplotypes (maternally inherited) were estimated based on the Median-Joining method using PopART 1.7 [34].

### Population genomic analyses

We measured the degree of genetic differentiation among populations by conducting analyses of population genetics and landscape genetics based on allelic and haplotype frequencies. We evaluated genetic structure using a principal component analysis (PCA) generated with the SNPRelate package version 3.3 [35] in R 3.5.2 [36] and with the STRUCTURE 2.3.4 [37] and Admixture 1.3.0 [38] programs, exploring values of K from 1 to 6. For the Admixture analysis we first thinned our dataset to avoid including linked markers by retaining 3,958 SNPs that were at least 10 kb apart, using VCFtools version 0.1.16 [39]. We compared models with different K values using the cross-validation method with 10 iterations. We chose to plot the results for K = 3 in the main manuscript as these are concordant with our other analyses. K values beyond this point did not show further structure and are displayed in the Supplementary Figures in S1 File. For the Structure analysis, we exported a single SNP per locus (retaining 4,527 SNPs), selected randomly using the populations Stacks module, to avoid including tightly linked SNPs. Each run consisted of 500,000 generations after 250,000 iterations were discarded as burn-in. We ran 10 iterations per value of K and did not incorporate sample location in the model. We implemented the admixture ancestry model with correlated allele frequencies and λ = 1 as the allele frequency prior. The level of genomic differentiation (between sectors: Pacific coast, Nahuel Huapi Lake, and Atlantic coast) was determined by calculating the F_ST_ values for each SNP, and by performing an AMOVA (analysis of molecular variance) with the *population* module in Stacks. We also used the *population* module to calculate heterozygosity, nucleotide diversity and inbreeding coefficients. We ran fineRADstructure v0.3 [40], which clusters individuals based on haplotype frequencies to determine population structure, conducting 100,000 iterations following 100,000 burn in repetitions (sampling every 1,000 iterations), and inferring a tree. We built a Maximum Likelihood phylogenetic tree in RaxML version 8.2.4 [41] after implementing the ASC_GTRGAMMA model and the Lewis correction for ascertainment bias and conducting 200 bootstrap replicates to assess node support. We used VCFtools to calculate relatedness values among individuals from the Nahuel Huapi Lake colonies. Finally, we estimated migration among geographic regions using BayesAss-SNPs 3.0.5.6 [42], running the program on the same SNP dataset used for the Admixture analysis, for 10 million generations following a burn in of four million generations. We fine-tuned the MCMC mixing parameters by using the following commands: -a0.23 -f0.01. We assessed convergence by inspecting traces and effective sample sizes in Tracer 1.7.1 [43].

## Results

**Fig 2:**
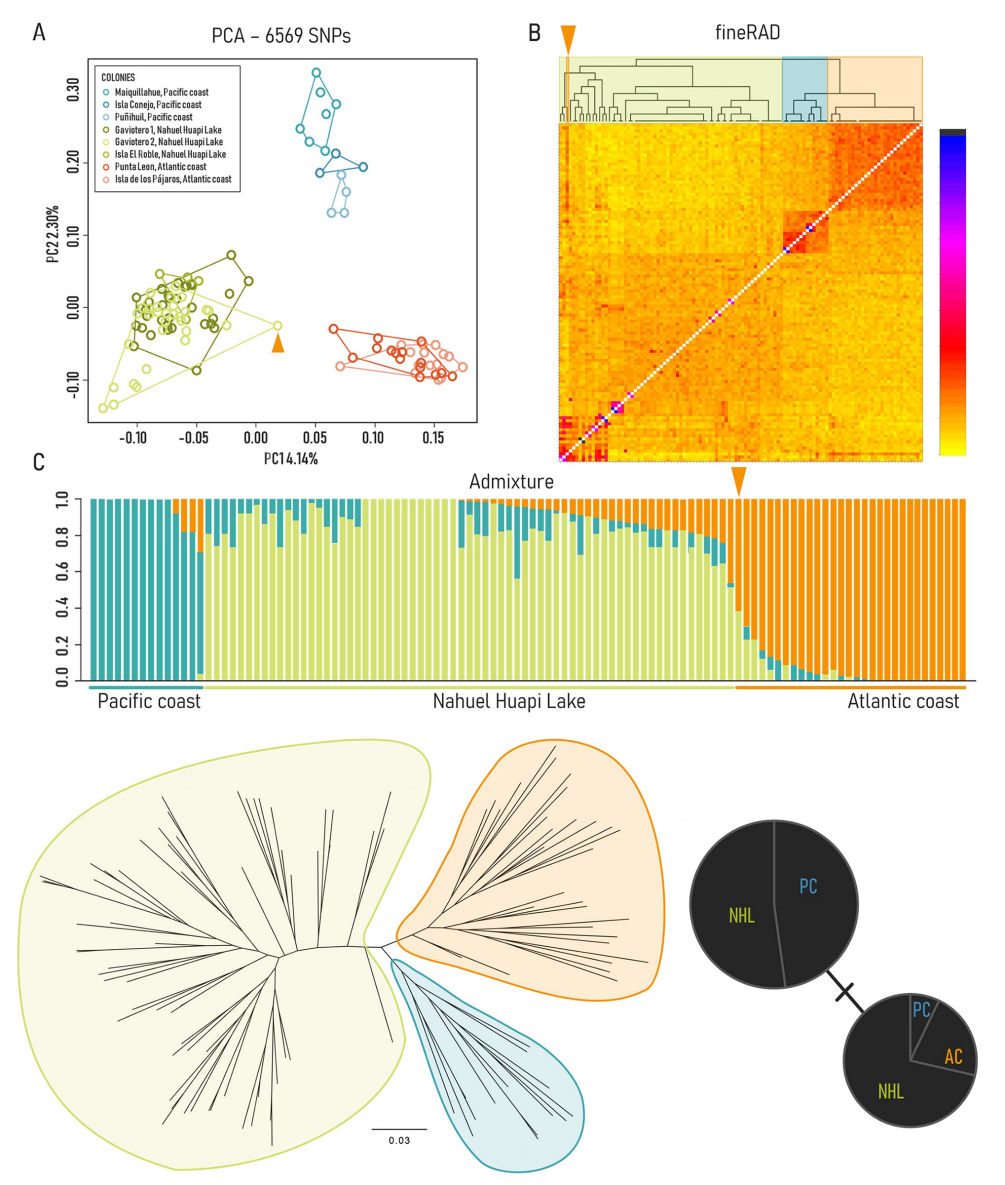
Population structure from Kelp Gulls collected in Nahuel Huapi Lake, the Atlantic and Pacific coast colonies. (A) Principal component analysis (PCA) based on 6,569 SNPs. Circles represent individuals and colors denote sites of origin as indicated in the legend. (B) FineRADstructure plot derived from haplotype data showing clustering according to geographic location. The matrix represents the co-ancestry values between all pairs of individuals, and the magnitude of these values is color-coded as shown by the scale-bar. The orange arrow denotes an individual sampled on the Atlantic coast with a large proportion of Nahuel Huapi Lake ancestry (C) Admixture plot of Kelp Gull populations (K = 3). Each individual is represented by a vertical bar broken into different colored genetic clusters showing the proportion of genetic ancestry assigned to each cluster. The orange arrow indicates the individual mentioned in (B). (D) Maximum Likelihood tree showing phylogenetic relationships between individuals from the three regions represented with three different colors as in (B). Node support for each of the main clades was 87 (PC), 61 (AC), 50 (NHL), and 50 (AC, PC). (E) Haplotype network generated from COI mitochondrial sequences.

We found individuals from the Pacific coast, Nahuel Huapi Lake, and the Atlantic coast belong to three genetically differentiated populations (F_ST_ and Phi_ST_: NHL vs. PC = 0.018 and 0.04, respectively; NHL vs. AC = 0.023 and 0.04; PC vs. AC = 0.027 and 0.03). The PCA (based on 6,569 SNPs) shows three distinct clusters corresponding to each one of the three geographic regions (Fig 2A). The first principal component (PC1) explains 4.14% of the total variance, separating the freshwater and coastal populations. PC2 explains 2.3% of the variance and separates the Pacific colonies from the remaining individuals, as well as resolving differences among these colonies (which did not occur for colonies in the remaining areas). In addition, the fineRADstructure results are consistent with the PCA, showing that individuals within the three populations cluster together (Fig 2B), as shown by the dendrogram above the inter-individual co-ancestry matrix. Within Nahuel Huapi Lake, we found groups of individuals that cluster together despite being sampled at different locations. While the average relatedness among NHL individuals is 0.01 (and, for comparison, 0.06 and 0.083 in AC and PC, respectively), the smaller clusters average 0.07 and 0.28, with pairs of individuals reaching relatedness values of up to 0.72 (Fig 2B). This suggests we sampled related individuals in these smaller breeding populations. The fineRADstructure and particularly the Admixture analysis (Fig 2C), show admixture among all three regions, with a lesser degree of Nahuel Huapi Lake ancestry in the Pacific coast. We obtained a similar result using Structure, and for both analyses values of K above 3 did not uncover additional population structure (S1 and S2 Fig in S1 File). The fineRADstructure analysis revealed an individual sampled on the Atlantic coast with Atlantic coast and Nahuel Huapi Lake ancestry, that clusters with other freshwater individuals, which is evidence of connectivity among colonies. Our phylogenetic analysis as well as the clustering analyses show that the three populations are similarly differentiated from each other, with populations from marine environments being slightly more closely related to each other than to the Nahuel Huapi Lake population (e.g., Fig 2B and 2D). We note that in agreement with the relatively low levels of differentiation among populations, our phylogenetic analysis produced generally low node support (Fig 2D). Overall, different measures of diversity were similar across the three regions, with the Nahuel Huapi Lake population showing the lowest heterozygosity and nucleotide diversity (Pi) (Table 1). Finally, the highest proportion of migrants was estimated from NHL into PC, followed by NHL into AC (Table 2). Gene flow into NHL from either PC or AC was comparatively much lower. Our mtDNA analysis did not provide sufficient resolution to differentiate populations, as we found two COI haplotypes among our samples, one of which was present in both the NHL and PC colonies, while the second was found in the three regions (Fig 2E).

**Table 1:** Summary statistics for different Kelp Gull (*Larus dominicanus*) populations. Observed (H_obs_) and expected (H_exp_) heterozygosity, nucleotide diversity (Pi), and inbreeding coefficient (F_is_).

	H_obs_	H_exp_	Pi	F_IS_
Nahuel Huapi Lake	0.248	0.233	0.235	-0.025
Pacific coast	0.267	0.252	0.262	-0.005
Atlantic coast	0.272	0.260	0.265	-0.012

**Table 2:** Estimates of gene flow among populations. Donating populations are shown in the column on the left while receiving populations are in the top row. Values represent the fraction of individuals in the receiving population that are migrants derived from the donating population (per generation). For example, ~30% of individuals from PC are estimated to be derived from NHL in every generation.

	NHL	PC	AC
NHL	0.983	0.297	0.056
PC	0.008	0.683	0.017
AC	0.008	0.015	0.924

## Discussion

This is the first genetic study to include freshwater Kelp Gull colonies from Patagonia, and we find that those from Nahuel Huapi Lake are genetically differentiated from their closest coastal Atlantic and Pacific counterparts. Previous studies on Kelp Gulls have found genetic structure both at a South American continental scale [22–24] and among populations along the Argentine coast [16]. Particularly, Lyons et al. [16] found some level of differentiation among the most geographically distant colonies in their study, as expected from a pattern of isolation by distance with gradually diminishing connectivity with distance along the Argentine coast. Our findings also indicate closer affinities among colonies within each specific region, as opposed to the relationships observed between colonies situated in distinct geographic regions. In this context, we initially expected higher connectivity between Nahuel Huapi Lake populations and those from the Pacific coast, given their proximity and the existence of potential ecological corridors across the Andes Mountains, compared to the Atlantic coast. Instead, our results show that coastal colonies are more closely related to each other than to those from freshwater, despite their considerable geographic separation. This generally low level of differentiation between Atlantic and Pacific colonies may be related to the continuous presence of numerous colonies along these coasts, with a gap in the southern region of Chile which may be due to a lack of information resulting from the difficulty of accessing and surveying this area [9]. However, this does not imply a lack of connectivity between coastal colonies and those from Nahuel Huapi Lake. First, our results show admixture between freshwater and coastal populations, primarily between NHL and AC. Moreover, we observed an adult individual sampled in the Isla de Los Pájaros colony (on the Atlantic coast) with a considerable proportion of genetic ancestry from NHL and the remaining ancestry from AC. This bird is likely the offspring of a Nahuel Huapi Lake migrant which bred with an Atlantic coast individual (although more complex scenarios of dispersal are also compatible with this finding). Additionally, during the course of this study, two records of juvenile individuals banded in Nahuel Huapi Lake were recovered in coastal localities, one was found on the Pacific coast (Osorno, Chile) and the other on the Atlantic coast (Rio Negro Province, Argentina), indicating gulls are capable of dispersing among geographic regions, although this is not necessarily indicative of breeding. Concordantly, our estimates of gene flow suggest migration from NHL into both PC and AC. We did not find direct records of individuals captured in the lake that originated from coastal colonies. Taken together, the generally low levels of differentiation observed in our ddRAD data, and the lack of differentiation in mtDNA, suggests a recent history of divergence, with ongoing gene flow among regions.

An evolutionary significant unit refers to a population or group of individuals that share genetic characteristics [1], and can evolve even in metapopulations of species due to factors such as philopatry [25, 26]. In this regard, our findings imply that the colonies in Lake Nahuel Huapi should be considered an evolutionary significant unit, separate from the coastal colonies. The growth of Kelp Gull populations in many of their coastal colonies is influenced by anthropogenic food subsidies [11]. The available information from colonies situated in Nahuel Huapi Lake shows that Kelp Gulls also use landfill sites as a food source both during and after the breeding season [13, 44]. There is a strong relationship between the areas utilized by seabirds during the winter and their dispersal movements, and this is associated with the existence of population structure [7]. As a consequence, the use of local anthropogenic food sources during the winter might explain the patterns of genetic clustering observed in our study. Furthermore, our results also suggest that the demography of these freshwater colonies is not directly dependent on the movement of individuals from nearby colonies, and thus the likely population growth of Kelp Gulls in Nahuel Huapi Lake is self-sustaining.

Another Patagonian seabird, the Imperial Cormorant (*Leucocarbo atriceps*) also has freshwater populations that are genetically differentiated, both in Lake Yehuin (Tierra del Fuego, Argentina) and in Nahuel Huapi Lake [45]. While the NHL population is likely derived from Pacific coast individuals, those from Lake Yehuin show stronger genetic differentiation [45]. The NHL population shows ecological differences with respect to other marine Imperial Cormorants [46], and those from Lake Yehuin are morphologically differentiated (showing smaller body size and nasal-glands), perhaps as a consequence of local adaptation [47]. It remains to be determined whether the genetically differentiated freshwater population of Kelp Gulls also shows specific adaptations to these environments that differentiate them from those found breeding on the coast.

Understanding the dynamics of Kelp Gull populations, which may function as sources, sinks, or occur in relative isolation from other colonies, has implications for conservation and disease ecology. The Kelp Gull, like other gull species [48–50], is a known carrier of pathogens and parasites in several areas along its distribution, some of which can cause diseases in humans. For instance, Kelp Gulls can be carriers of enterobacteria such as Salmonella [51, 52], influenza virus [53, 54], and helminths of the trematoda class (ex Schistosoma) that can cause dermatitis in humans. Recent reports have confirmed the presence of cestodes from the genus Diphyllobothrium in Kelp Gulls nesting in Nahuel Huapi Lake [55]. The eggs of these cestodes are released in the aquatic environments together with the feces of the definitive host (ichthyophagous birds and mammals, including humans), and can cause Diphyllobothriasis [56]. Furthermore, considering that this species can be problematic, negatively affecting other birds with which it usually nests [56], knowledge of the connectivity of its populations and the identification of management units can be fundamental tools when applying management and conservation measures. Our study is the first to identify a freshwater Kelp Gull colony from Patagonia as an evolutionary significant unit. However, to achieve a more complete understanding of the metapopulation dynamics and connectivity of this species as a whole, as well as the origin of freshwater colonies, it is necessary to expand the geographic scope of sampling and include more colonies across the species’ distribution.

## Supporting information

S1 File (PDF)
