# Supplementary material for: Population structure and connectivity among coastal and freshwater Kelp Gull (Larus dominicanus) populations from Patagonia
Source: PLoS One. 2024 Apr 18;19(4):e0301004. doi: 10.1371/journal.pone.0301004 (PMC11025793; doi:10.1371/journal.pone.0301004)
Supplement: S1 File — (PDF) [file pone.0301004.s001.pdf]

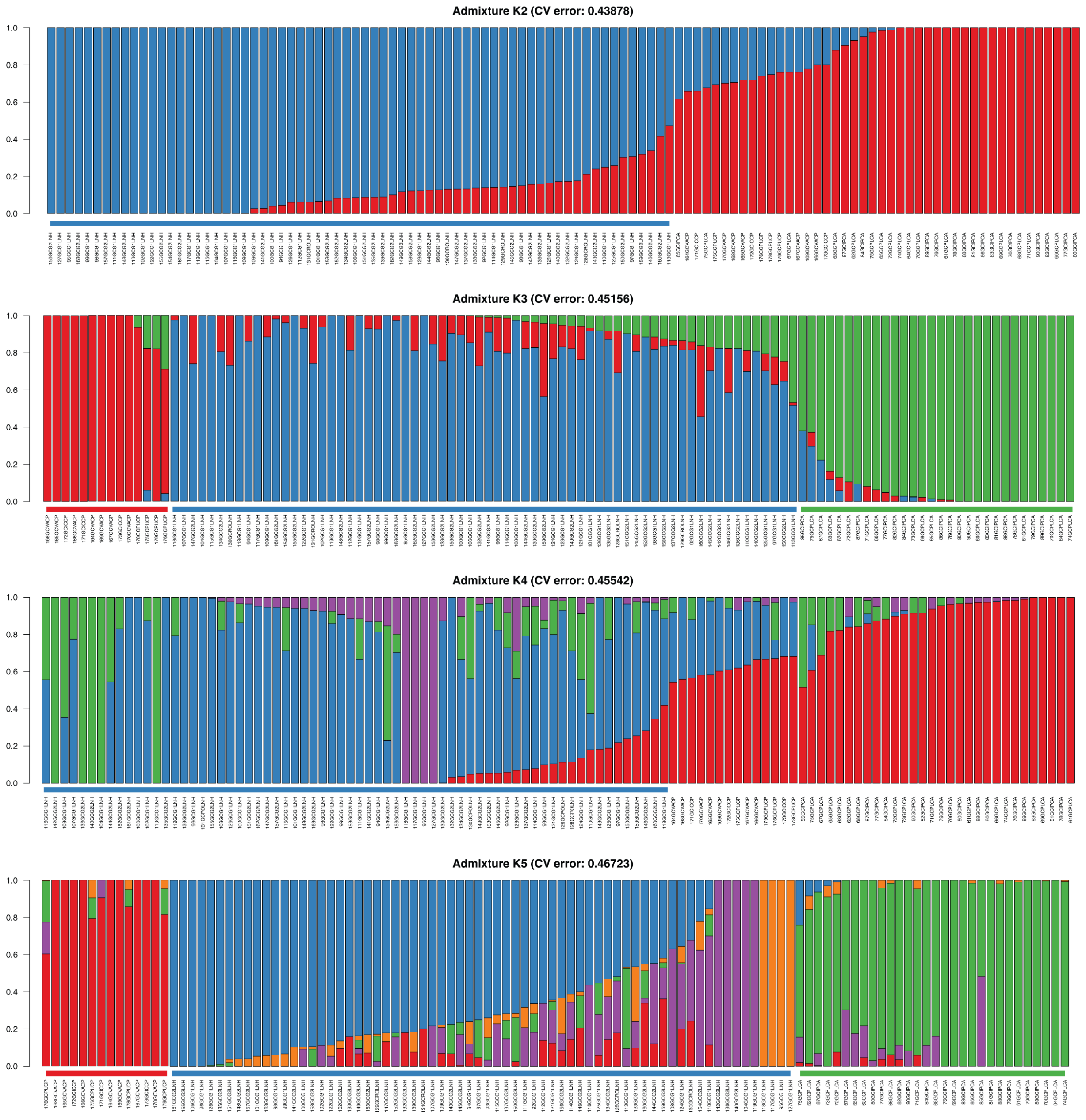

Figure S1. Results from Admixture runs (K=2 to K=5). Each plot shows the cross-validation (CV) error for that K value. We also explored values of K from 6 to 10, but these showed higher CV errors and did not identify further population structure. The horizontal bars above the sample names group individuals by geographic populations when they match with a genetic cluster. The Pacific coast is indicated in red, samples from Lago Nahuel Huapi are shown in blue and the Atlantic coast is in green.

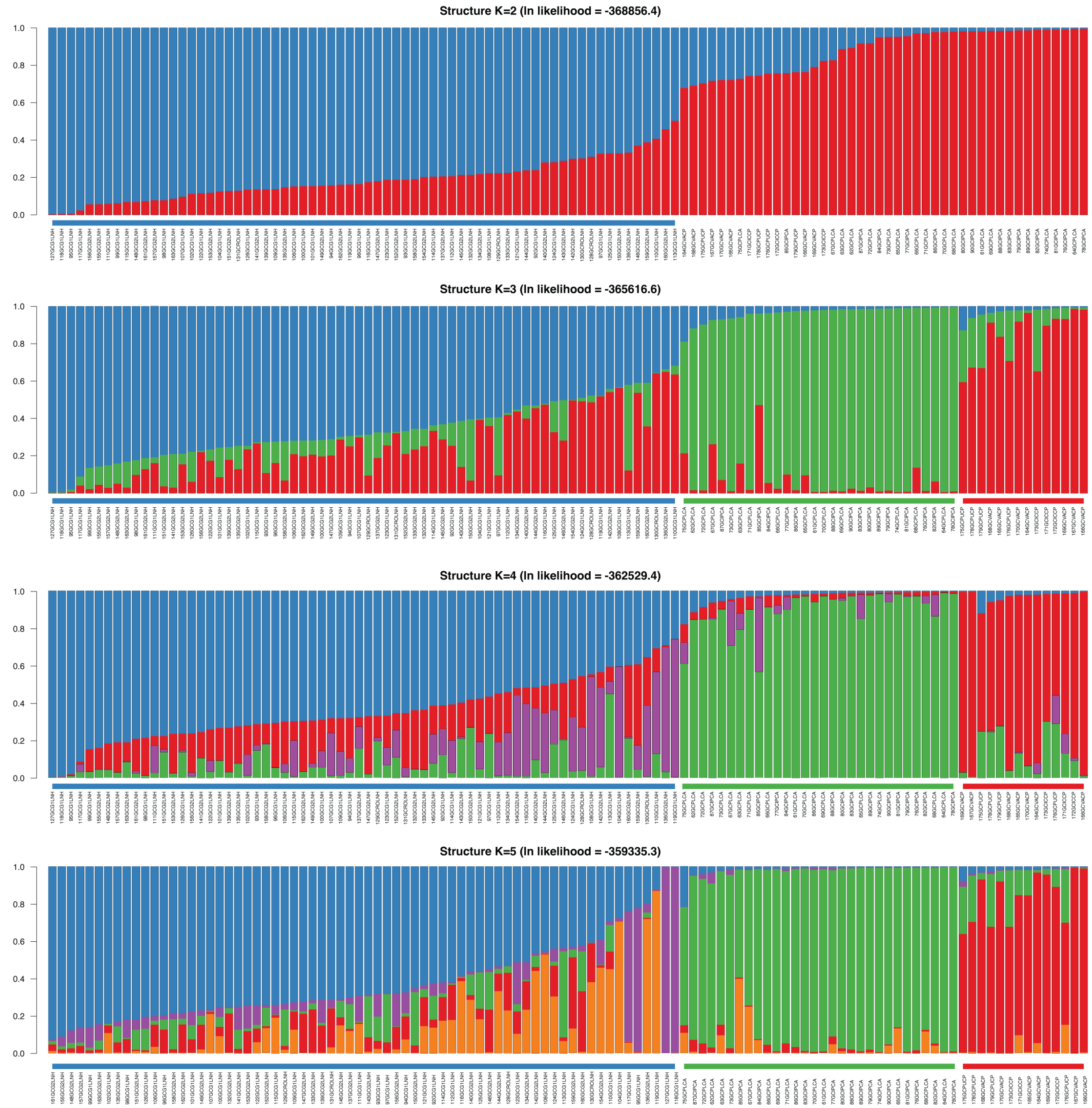

Figure S2. Results from Structure runs (K=2 to K=5). Each plot shows the ln likelihood for that K value. Other details as in Figure S1.
